# Close encounters: Interpersonal proximity amplifies social appraisals

**DOI:** 10.1111/bjop.12781

**Published:** 2025-02-21

**Authors:** Kristina Veranic, Andrew P. Bayliss, Mintao Zhao, Ian D. Stephen, Louise Ewing

**Affiliations:** ^1^ University of East Anglia Norwich UK; ^2^ Bournemouth University Poole UK

**Keywords:** body perception, face perception, interpersonal distance, person perception, trait attribution

## Abstract

Social appraisals reflect the rapid integration of available perceptual information with broader contextual factors (e.g., intentions). While interpersonal distance affects both information availability and social context, how it changes trait impressions remains unknown. Over four experiments, we used a novel paradigm to address this question. In Experiment 1, we assessed participants' attributions of attractiveness, competence, dominance and trustworthiness of life size full body images of people when they appeared at near (1 m) and far (4 m) distances. Proximity amplified the relative magnitude of both positive and negative socio‐evaluative impressions. However, this effect of proximity leading to more extreme positive or negative ratings was selectively weaker for aesthetic (attractiveness) judgements. In Experiment 2 (size) and Experiment 3 (spatial frequency), we held distance constant while manipulating visual cues relating to implied distance, revealing broadly similar results to Experiment 1. In Experiment 4, we used the interpersonal comfort distance paradigm to confirm that our life‐sized projected images elicited similar comfort distance to interacting with a real person, helping to validate our general approach. These findings demonstrate the crucial role of interpersonal distance in impression judgements.

## BACKGROUND

Social interactions occur at specific (real or virtual) distances between individuals. Our perception and regulation of ‘comfortable’ interpersonal distances are influenced by a range of factors (e.g., gender, personality, context) and constitutes an important social signalling mechanism (e.g., via approaching versus avoiding; Candini et al., 2021; Iachini et al., 2016; Perry et al., 2013). The space between people also constrains the type and quality of sensory information that is available for different social judgements. Visual inputs, for example, can differ substantially when we are talking to someone close to us versus farther away (e.g., spatial frequency information, proportion of the body visible). Thus, a comprehensive understanding of the mechanisms underlying social evaluation warrants targeted consideration of how such *spatial* information affects social interaction. Across a series of experiments, the current study investigates the role played by interpersonal distance in a variety of trait judgements to better understand how spatial context influences the operation of person perception processing in the real world.

Previous research suggests that there may be an inherent link between spatial and social cognition. Trait impressions are fast spontaneous judgements people make about others based on their appearance, which often remain stable over time and there is a high consensus in the ratings among individuals (Oosterhof & Todorov, 2008; Sutherland et al., 2013; Willis & Todorov, 2006). The judgements made by individuals are significantly influenced by both the person making the judgement and the context in which they find themselves (Funder, 1995). Similarly, it has been shown that personality affects how people maintain and perceive distances from others in social interactions. For example, people high in trait dominance and social class are observed to keep smaller distances to others in social interactions (Hall et al., 2005). In contrast, people high in social anxiety have been found to overestimate closeness of another person, leading them to maintain larger interpersonal distances (Givon‐Benjio & Okon‐Singer, 2020; Perry et al., 2013). Furthermore, people experience lower empathy towards others that are further away or separated from them by a barrier (Schiano Lomoriello et al., 2023).

The context of social interactions also affects how people regulate interpersonal distances. For instance, from an evolutionary perspective it is advantageous for people to keep greater distances when perceived threat is higher (e.g., individuals with angry or disgusted expressions), when encountering approaching individuals, (Ruggiero et al., 2017; Vieira et al., 2017) or when others act unfairly or immorally (McCall & Singer, 2015). Furthermore, more intense reactions are more likely in close proximity, whether negative because of the reduced ability to take evasive action or positive because of the increased chance of an intimate encounter.

Body shapes and postures are important sources of information for making social attributions about others (Hu et al., 2020; McElvaney et al., 2021). For example, looking to identify principal components of trait impressions from body shapes, Tzschaschel et al. (2022) found that body trait impression dimensions are best described by two dimensions–trustworthiness and dominance, in line with those made from faces. Hu and O'Toole (2022) asked participants to evaluate photographs of people with sometimes obscured faces or bodies, on a range of different personality traits. Their results indicated that some social evaluations appear to be made primarily from the face‐based information (e.g., trustworthiness), others are based on body information (e.g., self‐discipline), but most reflect the integration of facial and bodily information. In real life, one's consideration of socially relevant information available in the face and body is critically dependent on interpersonal distance. For instance, Hahn et al. (2016) studied the contribution of the face and the body to person recognition across distances. They found that with decreasing distance the reliance on the body decreases and the reliance on the face increases, when both are present. At substantial distances, the relatively lower spatial frequency of a face on the retina will interfere with a detailed examination of features that is easier when it is close enough to be fully resolved. In contrast, a person's whole‐body information is not visually available at very close distances. Additionally, the body is a larger, coarser stimulus wherein fine‐grained detail is less perceptually useful. Impressions derived from the body are therefore likely to be more resilient to the loss of high spatial frequencies at further distances.

Despite its crucial role in social perception and interaction, interpersonal distance has rarely been considered in person perception research investigating social attributions, which have been typically focused on understanding the relative importance of traits across the general population, rather than individual differences in the perception thereof. Such work predominantly asked people to judge social and personality traits based on small‐size photographs shown on a computer screen, which can imply a larger distance than is typical in everyday social interactions. The limited extant work on this topic suggests that space information is relevant for the processing of trait attributes. For instance, Patterson and Sechrest (1970) asked participants to form impressions (aggressiveness, friendliness, extraversion, dominance) of confederates sitting at set distances from them (0.60 m, 1.20 m, 1.80 m, 2.40 m) during an interview and found that trait ratings generally decreased with distance, with the exception of somewhat lower ratings at the closest measured distance. They attributed the negative linear trend to people appearing more ‘socially active’ at closer distances. Bryan et al. (2012) presented photographs of the same face identities taken at 0.46 m versus 1.37 m distance (i.e., within versus outside participants' estimated personal space, respectively). They observed lower ratings of social traits (trustworthiness, competence and attractiveness) for the images captured within the estimated personal space. Across a series of experiments, a recent study by Trifonova et al. (2024) investigated judgements of trustworthiness and dominance with videos of avatars standing near/far, approaching/receding, camera moving towards/away from the avatars. They found higher ratings of both traits when avatars were approaching in comparison with standing still, however no differences were found between close and distant images. They also found that ratings of dominance were higher when avatars were approaching or the camera approached them; meanwhile trustworthiness ratings were higher during the movement of the avatars compared with when they stood still, even when the camera was moving towards them. They conclude that the movement of the avatars appears more naturalistic compared with stillness and that towards motion increases ratings of dominance and speculate that distance and dynamic cues might have interacted when forming these impressions. These studies suggest that physical or implied interpersonal distance can have consequential effects on trait judgements.

### Present study

Given that encounters with others at near versus far distances contain different sensory information and afford different types of social interactions, we aimed to explore whether differences may exist when people form their impressions of others appearing at relatively near and far interpersonal distances. We conducted four experiments to investigate how interpersonal distance contributes to social perception of others in an ecologically valid setting. Experiment 1 served as our principal investigation of the effects of interpersonal distance on impression formation. Here we asked participants to rate the perceived competence, dominance, attractiveness and trustworthiness of life‐sized images of other people standing near versus far away. Experiments 2 and 3 examined whether the influence of distance on impression is mediated by distance‐related lower‐level perceptual information: object size (Experiment 2) and spatial frequency information (Experiment 3). Spatial frequency filtering is the removal of certain portions of spatial frequency information from the original image. Images with high spatial frequency information retain information about edges and details, which has been related to the processing of the fine‐scale local information in faces. Low spatial frequencies retain global information, which have been related to the processing of the large‐scale global information in faces (Goffaux & Rossion, 2006). Images retaining low spatial frequencies visually represent stimuli at larger distances from the observer (see Loftus & Harley, 2005). Finally. Experiment 4 investigated whether participants maintain ‘comfort distances’ to life‐size image presentation of people consistent with those observed with real people. This experiment also served to help validate our novel approach to the study of interpersonal distance in this context. Together, these experiments aimed to answer two main questions: (1) do our impressions of others differ when they appear near versus far away? (2) What visual properties of stimuli underlie the influence of interpersonal distance on social perception of other people?

The limited prior research relating distance with trait impressions has used either implied distance (Trifonova et al., 2024) or distance‐related cues (Bryan et al., 2012) or has tested how personality traits of a small number of confederates (judged after an extended interview) are made across different distances (Patterson & Sechrest, 1970). In our study, we wanted to assess appraisals of a wide range of realistic social stimuli, while maintaining high levels of experimental control. We therefore opted to display life‐sized, whole‐body photographs of participants on a projector screen to be rated at two interpersonal distances. ‘Near’ stimuli were presented at a distance of 1 m from participants. This distance reflects the average space that the local (English) population prefers to keep from strangers (Sorokowska et al., 2017) and corresponds to the region of ‘personal space’ where the majority of social interactions are likely to occur (Hall, 1963). Furthermore, this distance of 1 m allowed for the body to be visible and thus could contribute to person perception. In contrast, ‘far’ stimuli appeared 4 m away. At this range, the social relevance of an individual is much lower and they are considered to be in ‘public space’ (Hall, 1963), where the resolution of visual information is also lower than at more proximal distances.

We tested four trait dimensions commonly considered in the first impressions literature: trustworthiness, attractiveness, competence and dominance (e.g., see Oosterhof & Todorov, 2008; Sutherland et al., 2020, for review see Sutherland & Young, 2022). In our experiments, we focus on the individual differences when making these social judgements and the conditions under which they can vary. Due to the lack of research on this topic our hypotheses were exploratory. We broadly hypothesised that ratings of all these traits would differ between the near and far distances and also that there might be variability in the magnitude of effects observed across traits. Our prediction was not necessarily that judgements made at near and far distances would be driven by distinct mechanisms, but that such stimulus ratings might nevertheless vary, e.g., due to differences in the visual information available.

## EXPERIMENT 1

In Experiment 1, we investigated how interpersonal distance modulates high‐level social perception of others. We presented life‐sized images of people and asked participants to rate them for trustworthiness, competence, dominance and attractiveness when standing at a near (1 m) versus far (4 m) interpersonal distance. Given that small‐sized photographs used in most previous studies imply a far interpersonal distance, we treated ratings at far distance as baseline and tested how being more proximal to people affects impressions. We hypothesised that varying proximity would modulate impressions–and that effects might vary between different traits, particularly if the effects are driven by socio‐evaluative considerations rather than a more general mechanism (e.g., low‐level differences in visual information).

### Method

#### Participants

Sixty participants (*M* = 30.4 years, *SD* = 17.3 years, range from 18 to 47 years; 42 female, 16 male, 2 non‐binary) completed this Experiment. One participant was excluded from the data analysis due to poor engagement. The sample size was chosen to match similar studies of first impressions (e.g., Oosterhof & Todorov, 2008).

For this and subsequent experiments reported here, participants provided informed consent, and the procedure of the study was approved by the local Ethics Committee (reference code: ETH2324‐0775). Participants in our studies received course credits or a small monetary compensation for participation. The majority of the participants were Psychology undergraduate students, the remainder were from the local community.

#### Stimuli and apparatus

**FIGURE 1:**
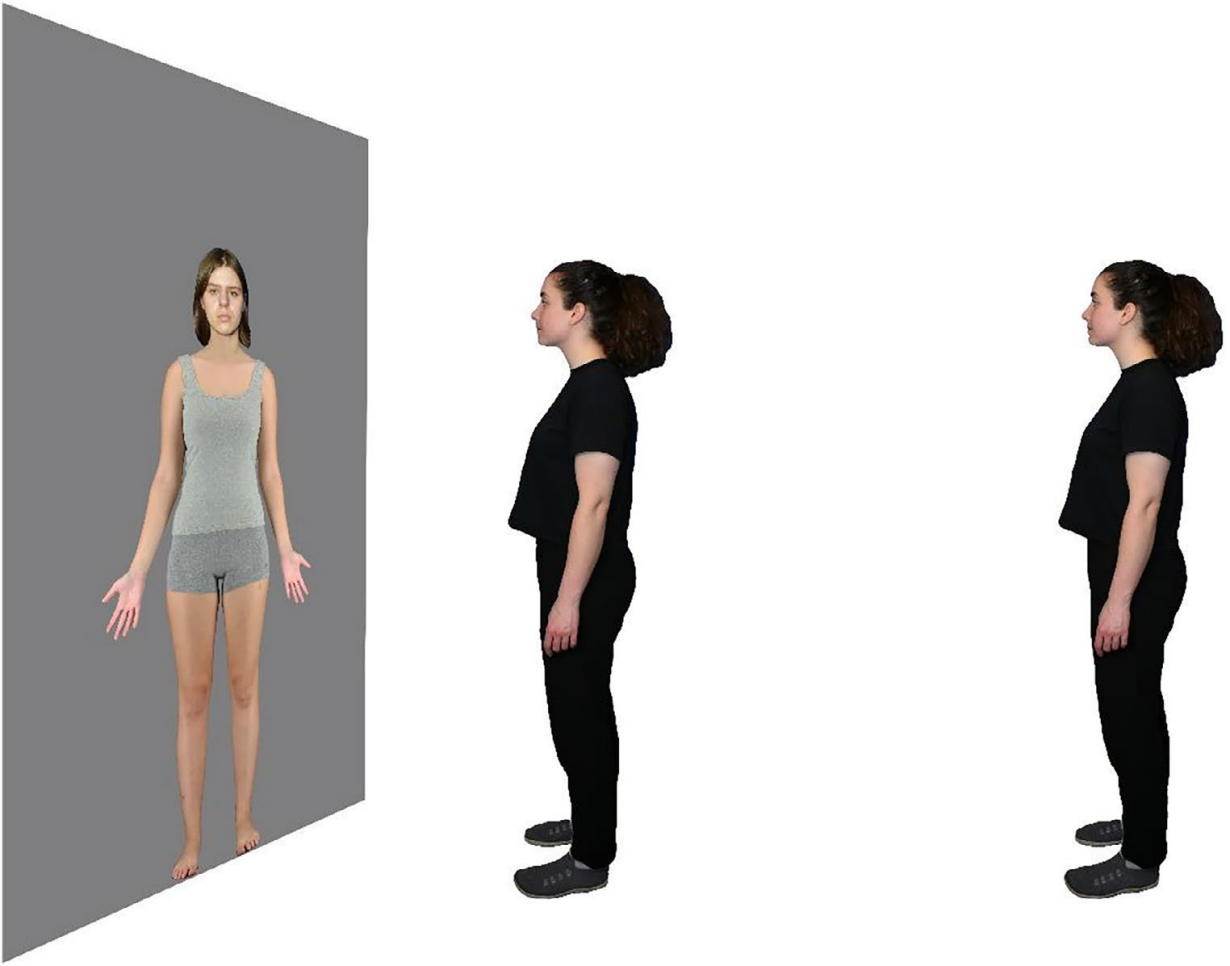
Schematic representation of experimental apparatus for Experiment 1. Participants stood either 1 m (near) or 4 m (far) from the screen. Images were rear projected onto the screen to allow the participants to stand at near distances without obstructions. The rating scale appeared above the image. Participants made the ratings with a computer mouse placed on a stand next to them.

This and subsequent experiments reported here were programmed using Gorilla Experiment Builder (Anwyl‐Irvine et al., 2020). The stimuli consisted of high resolution (1794 × 4494 pixels) images of 96 adults of different ages and ethnic backgrounds from an existing database (for more detail see Stephen et al., 2016). Individuals are pictured wearing standard close‐fitting grey singlets and shorts and facing forward in a standard posture–with their arms by their side–and a neutral facial expression (Figure 1). Each image was positioned on a grey background, so that the individual appeared to be approximately standing on the same ground plane. They appeared at realistic life sizes: males at a standard UK average height of 1.75 m, and females 1.60 m (NHS, 2019).

From the available stimulus set, we selected high and low‐rated exemplars for trustworthiness, dominance, competence and attractiveness based on pre‐ratings we collected online. The images were four, non‐overlapping stimulus sets (one for each trait). Here, the high and low bins comprised equal numbers of male and female stimuli (12 of each, 96 images in total), but were not matched for any other demographic variable (see Data S1 Supplementary Materials Section 1 for details on stimuli selection; see also http://doi.org/10.17605/OSF.IO/KJ6YC for demographic details of selected stimuli identities).

Images were rear‐projected onto a 2.3 m (height) × 1.5 m (width) white screen (Optoma GT1080 projector, 2800 Lumens (ANSI), working resolution of 1080 × 1920 pixels). This projector frame and set up was the same for all experiments reported here. Near‐distance images subtended vertically 84° of visual angle, and far‐distance images 25° when participants stand from 1 to 4 meters away respectively (see Figure 1).

#### Design and procedure

Participants were asked to stand at near (1 m) and far (4 m) locations (marked on the floor) from a projector screen onto which life‐sized images of people were rear‐projected. We measured participants' self‐reported arousal of the selected distances (see Supplementary Materials S2). Detailed descriptions of characteristics were given just before the trial started. Images appeared for 2500 ms; as soon as the image appeared, the participants could take as long as they needed to make their rating using a Likert scale (ranging from 1, meaning low on a trait–7, meaning high on a trait). They were asked to use the whole range of the scale, which was placed at the top of each image. They made ratings using a computer mouse on a stand next to them. Their rating was followed by a 500 ms interstimulus interval. Each participant rated all four traits in separate blocks (order randomized, with a new order for each distance block; order of stimulus gender was randomized within each trait).

#### Data analysis

We applied mixed effects models to investigate the modulation of distance on trait impressions. This approach allowed us to also consider any effects of variability associated with individual stimuli and participants, along with our fixed effects of interest. Initially the model used ANOVAs to examine the effects of *trait* (attractiveness, competence, dominance and trustworthiness), ‘*baseline stimulus ratings*’, which served to index impressions of each stimulus identity at one of the distances (we opted to use far distance ratings, given that these are most comparable to extant research in terms of stimulus size/implied distance), along with their *interaction* on distance‐related modulation of impressions (calculated as a difference score: far ratings minus near ratings for each item/stimulus). We examined these factors in a single model to estimate their overall contribution to distance‐related modulation of impressions. To account for potential variability associated with individual stimuli and participants, these were specified as random factors in the model.

To examine how distance affected trait ratings and to compare the effect of distance across traits, linear mixed effects models were produced for each trait, with distance‐related modulation of impressions (i.e., the difference between near and far ratings) serving as the outcome variable and baseline stimulus ratings (i.e., far rating) serving as the predictor variable. Participant and stimulus were again specified as random factors in the model. We then ran linear mixed effects models for each trait separately, to compare the relationships between baseline stimulus ratings and the distance‐related modulation of impressions between traits. Participant and stimulus were again specified as random factors in the model. Confidence intervals were compared to establish whether the traits were different to each other (non‐overlapping 84% confidence intervals can be considered equivalent to significance at the 5% alpha level; Payton et al., 2003). The 84% confidence intervals of the coefficients of the slopes were estimated using 1000 bootstraps (bias‐corrected accelerated method). Statistical analyses were carried out using RStudio 2023.06.1 (RStudio Team, 2020); with packages: ggplot2 (Wickham, 2016), dplyr (Wickham et al., 2023), tidyverse (Wickham et al., 2019), ez (Lawrence, 2016), resdr (Wickham et al., 2024a), tidyr (Wickham et al., 2024b), lme4 (Bates et al., 2015), cowplot (Wilke, 2020), sjPlot (Lüdecke, 2021), effects (Fox, 2003), lmerTest (Kuznetsova et al., 2017), multcomp (Hothorn et al., 2008), lmeresampler (Teh & Johnson, 2022), emmeans (Lenth, 2022) and extrafont (Jiminy, 2016).

### Results

Both the overall model and the separate models for each of the traits indicate that as baseline impressions of each stimulus increases (i.e., an individual received higher ratings at the far distance), the distance‐related modulation is increased for all traits. People's baseline ratings of all traits were more extreme at close distances. Importantly, modelling of different traits separately revealed that the effect of distance on attractiveness ratings is significantly different to all the other traits. The estimate of attractiveness in the model is less positive than the other traits, which means that people's impressions of attractiveness are more stable across distances than are their impressions of the other three traits. While being more proximal to an image of a person results in more extreme ratings of all traits, this effect is weaker for attractiveness.

The mixed effects modelling analysis revealed significant effects of trait, F(3, 83.88) = 9.15, *p* < .001, baseline stimulus ratings, F(1, 96.68) = 704.56, *p* < .001 and their interaction, F(3, 78.19) = 8.01, *p* < .001. The model shows that distance‐related modulation of impressions is significantly predicted by trait (i.e., if someone is considering attractiveness, trustworthiness, competence or dominance), baseline stimulus ratings (i.e., the extent to which an individual stimulus has high or low ratings on the given trait irrespective of distance) and their interaction.

**FIGURE 2:**
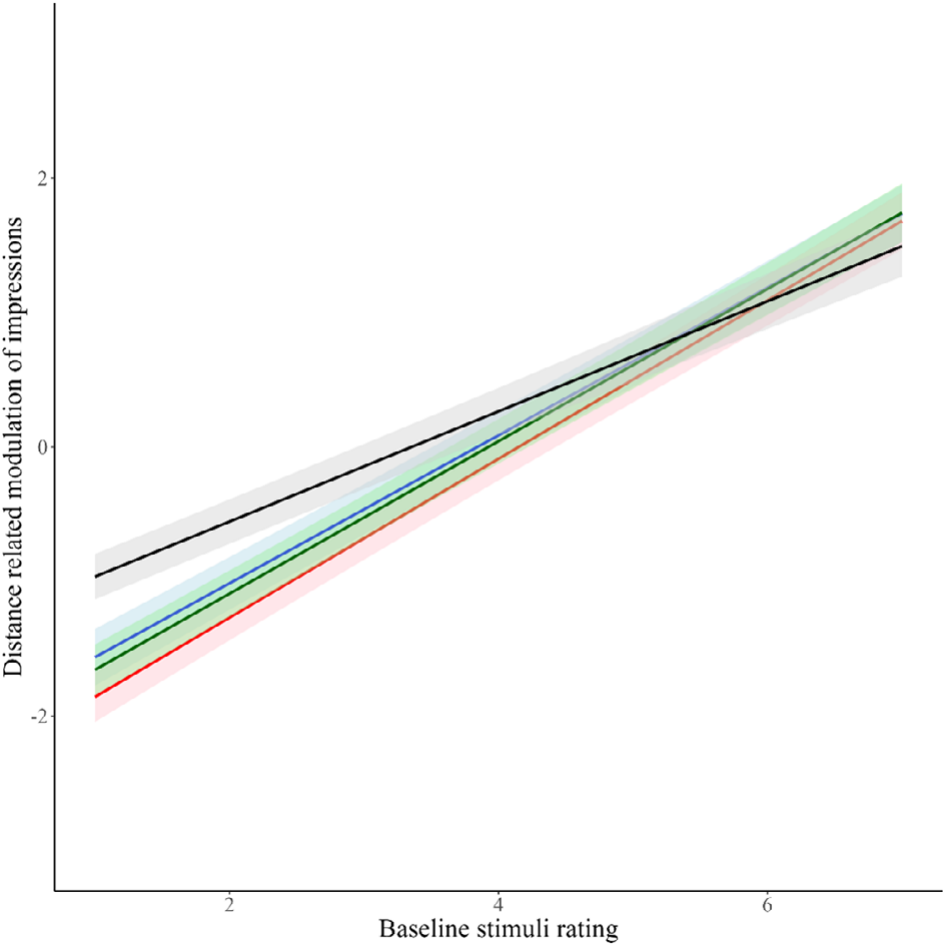
Relationship of distance‐related modulation of impressions and baseline ratings for each of the four traits. Black line = attractiveness; Red line = competence; Blue line = dominance; Green line = trustworthiness. Distance‐related modulation of impressions was calculated as Near minus Far, which means that the stimuli identities that were above the x = 0 were rated higher at Near and those below were rated as higher at Far distances.

Figure 2 depicts the relationship between distance‐related modulation of impressions and baseline stimuli ratings. The relationships for individual traits are presented numerically in Table 1. The positive slopes (*β*) indicate a positive relationship between distance‐related modulation of impressions and baseline stimuli ratings. One sample *t*‐tests show that in each case the slope is significantly different from zero. Together, these results show a substantial increase in the distance‐related modulation of impressions as baseline ratings increased. This means that increasing interpersonal proximity amplifies socio‐evaluative impressions (e.g., trustworthy looking people look even more trustworthy near, while untrustworthy‐looking people look even less trustworthy up close). This effect was qualified by the interaction between trait and distance‐related modulation of impressions, showing that attractiveness was less affected by distance than all the other traits. That is, highly attractive people are judged relatively similarly whether judged up close or more distally.

**TABLE 1:** Distance‐related modulation of the four traits.

Trait	*β*	*SE*	*84% CI*		*One sample t‐tests*
			*LL*	*UL*	
Attractiveness	.41	.02	.36	.45	*t* (25.59) = 18.23, *p* < .001
Competence	.59	.03	.53	.64	*t* (27.02) = 21.48, *p* < .001
Dominance	.55	.02	.50	.59	*t* (28.09) = 22.49, *p* < .001
Trustworthiness	.57	.03	.52	.61	*t* (27.19) = 20.55, *p* < .001

*Note*: *β* (*SE*) represents the mean (and standard error) of the slope for each trait. Confidence intervals (CI) are used to compare the differences in the slopes, where non‐overlapping 84% CIs indicate a significant difference at *p* < .05. *T*‐tests are used to determine if the slope is significantly different from 0.

Comparing the confidence intervals of the traits (presented in Table 1), we found the distance‐related modulation is different for attractiveness compared with the other traits–people's ratings of attractiveness were more similar at near and far distances compared with the other three traits. The positive relationship observed is significantly less steep for attractiveness compared to the three other traits.

Table 2 provides descriptive statistics detailing the stimulus ratings on each of the four targeted traits.

**TABLE 2:** Mean values and 95% confidence intervals of the four traits at Near and Far distances.

	Near (1 m)	Far (4 m)
Attractiveness	3.51 [3.43, 3.60]	3.57 [3.49, 3.66]
Competence	4.15 [4.07, 4.22]	4.11 [4.04, 4.18]
Dominance	3.83 [3.75, 3.91]	3.80 [3.72, 3.89]
Trustworthiness	3.94 [3.86, 4.01]	3.92 [3.85, 3.99]

### Discussion

The results of this experiment reveal that impressions of others are systematically amplified with increased proximity (images of people rated high/low on a trait far away are rated even higher/lower when rated near). Average ratings of an image scoring low on a trait at a far distance will on average score even lower when rated at a close distance, and a stimulus appearing high on a trait when rated at a far distance will on average be rated even higher when rated up close. This modulatory effect of distance operates differently across traits. Specifically, this amplification effect with proximity is detected but significantly weaker for attractiveness than for competence, dominance and trustworthiness. This may be because attractiveness judgements involve both socio‐evaluative and aesthetic (i.e. beauty abstracted away from social value common to social and non‐social perceptual experience) elements (see Saegusa & Watanabe, 2016). This finding implies that relatively more socio‐evaluative (rather than aesthetic) judgements are more affected by distance. One possible explanation is that judgements of trustworthiness relate to potential positive or negative interaction outcomes, which become more likely and consequential and proximal distances. However, the aesthetic elements of attractiveness judgements are stable across distance as they relate to a passive inherent quality of the image rather than to something the image/stimulus might be able to do. Another interesting possibility is that people may rely more on facial information to make judgements of competence, dominance and trustworthiness, about which information is carried with high spatial frequency information from the face and thus is less available from afar. It is possible that attractiveness cues could be drawn dynamically from the face and the body, with body information being more informative at greater distances (Honekopp et al., 2007; Hu & O'Toole, 2022; Thornhill & Grammer, 1999).

## EXPERIMENT 2

In Experiments 2 and 3, we tested the extent to which the observed effects of interpersonal distance on trait attribution in Experiment 1 might be driven by two perceptual cues to distance perception: visual size (Experiment 2) and spatial frequency information (Experiment 3). In Experiment 2, we manipulated stimulus size as a proxy for viewing distance and examined whether the effect of size change mirrors that observed in the distance manipulation of Experiment 1. To this end, we kept viewing distances constant at the close (1 m) and simulated the visual appearance of a stimulus at the far distance (contrasted with near distance) by presenting stimuli at a smaller size: matching the visual angle of the far stimuli in Experiment 1. If the effect of distance on trait perception is mediated by perceptual information like stimulus size, we would expect a similar pattern of responses as observed in Experiment 1. In contrast, if the mechanism of the effect of distance is social in nature, then changing stimulus size will not have the same influence on impression as physically moving close or away from other people.

### Method

#### Participants

Thirty‐two participants (*M* = 24.38 years, *SD* = 6.04, aged from 18 to 33 years; 23 female, 9 male, 0 non‐binary) completed the experiment. This sample size was used due to evidence that that 31 participants are sufficient to obtain stable averages for the targeted traits (95% confidence at +/− 0.50 values on a 1–7 Likert scale; see Hehman et al., 2018).

#### Stimuli

**FIGURE 3:**
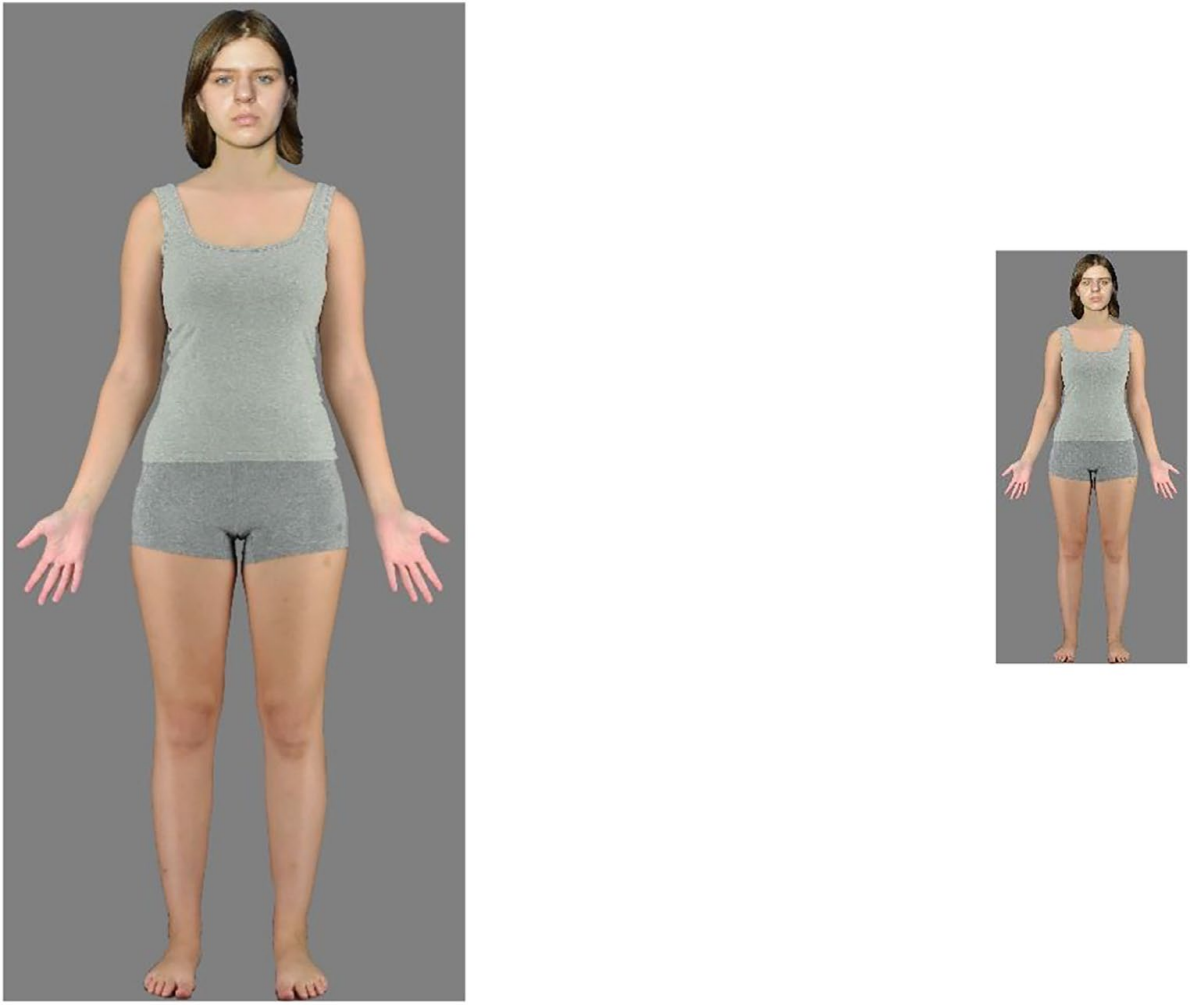
Representative examples of the Large and Small stimuli presented during Experiment 2. The stimuli presented in the experiments were all direct full body photographs of volunteers from Stephen et al. (2016). The authors hold permission from the volunteers to use their identifiable likelessess in studies but not in publication. Therefore these image are composites created solely for purposes of representing the stimuli. The body image was used in the experiment and the face is of an individual that was not used in the study but from whom we have permission to publish.

The stimulus set was the same as described in Experiment 1. Here the ‘large size’ images matched the life‐size images used in Experiment 1. The ‘small size’ images were reduced to match the visual angle of images presented at the far distance in Experiment 1–heights 26° for men (0.46 m) and 22° for women (0.38 m) (see Figure 3).

### Procedure

Participants stood a fixed 1 m from the projector screen and rated the same four traits for images of people when presented with the large and small sizes in separate blocks. All other aspects of the procedure were the same as in Experiment 1.

### Results

We conducted the same data analyses as reported for Experiment 1, using image size (large, small) as a proxy for distance (near, far) and using rating of small‐size stimuli as the baseline condition. Both the overall model and the separate models for each of the traits indicate that as baseline stimulus ratings increase (i.e., more extreme ratings), the size‐related modulation is increased i.e., people's baseline ratings of all traits were amplified (i.e., ratings were more extreme) with large images. This means that increasing image size amplifies first impressions (e.g., trustworthy looking people look even more trustworthy when large, untrustworthy looking people look even less trustworthy when large). Modelling of the different traits separately revealed that trustworthiness ratings are significantly different to all the other traits. The estimate of trustworthiness is more positive than the slopes of the other traits, which means that people's impressions of trustworthiness are relatively more amplified with larger images.

The mixed effects modelling analysis revealed significant effects of trait, F(3, 75.30) = 3.52, *p* = .018, baseline stimulus ratings, F(1, 51.14) = 488.26, *p* < .001 and their interaction, F(3, 68.26) = 4.89, *p* = .004. The model shows that size‐related modulation of impressions is significantly predicted by trait (i.e., if someone is considering attractiveness, trustworthiness, competence or dominance), baseline stimulus ratings (i.e., the extent to which an individual stimulus has high or low ratings on the given trait irrespective of distance) and their interaction.

**FIGURE 4:**
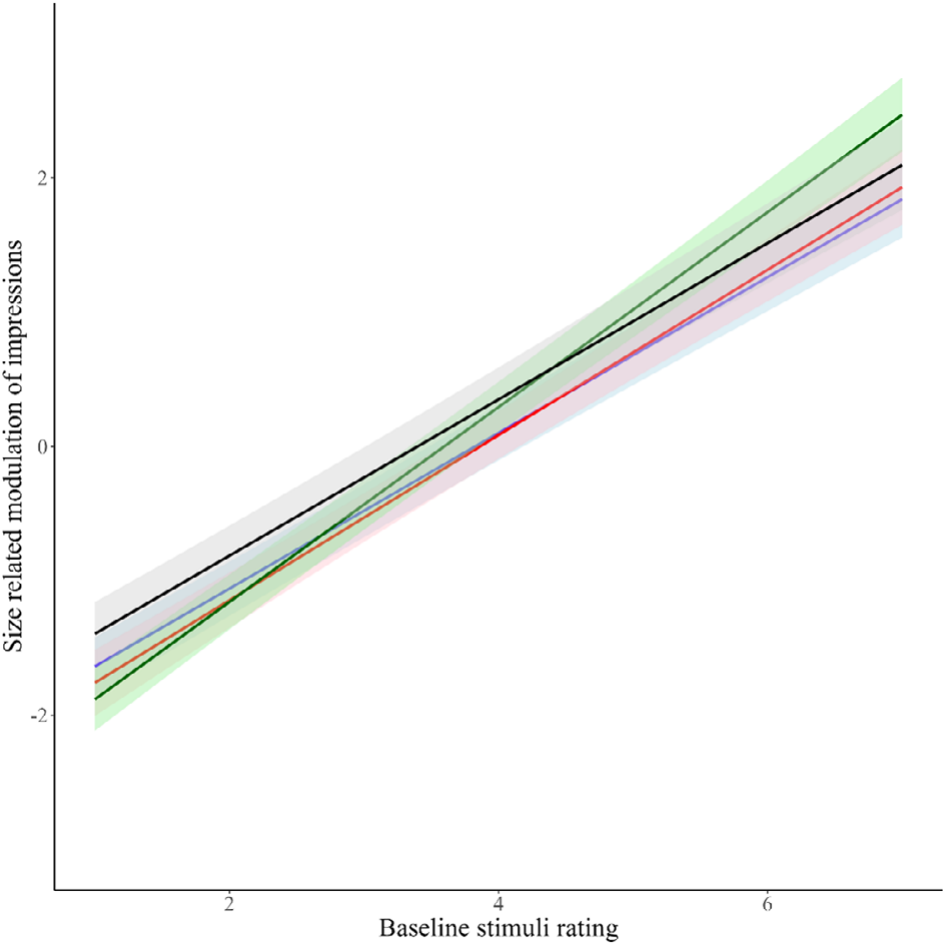
Relationship of size‐related modulation of impressions and baseline ratings for each of the four traits. Black line = attractiveness; Red line = competence; Blue line = dominance; Green line = trustworthiness. Size‐related modulation of impressions was calculated as Large minus Small, which means that the stimuli identities that were above the x = 0 were rated higher with Large images and those below were rated as higher with Small images.

Figure 4 shows the ratings of each participant of each stimulus for the four traits. It depicts the relationship between size‐related modulation of impressions and baseline stimuli ratings. These relationships are also presented in Table 3. The positive slopes (*β*) indicate a positive relationship between size‐related modulation of impressions and baseline stimuli ratings. One sample *t*‐tests show that the slope is significantly different from zero. Together, these results show a substantial increase in the size‐related modulation of impressions as baseline ratings increased. This effect was–as in Experiment 1, qualified by the interaction between trait and distance‐related modulation of impressions, showing that trustworthiness was more affected by size than all the other traits.

**TABLE 3:** Size related modulation of the four traits.

Trait	*β*	*SE*	*84% CI*		*One sample t‐test*
			*LL*	*UL*	
Attractiveness	.58	.04	.52	.63	*t* (25.47) = 15.31, *p* < .001
Competence	.62	.04	.55	.67	*t* (24.53) = 13.70, *p* < .001
Dominance	.58	.03	.53	.62	*t* (27.65) = 16.71, *p* < .001
Trustworthiness	.72	.04	.67	.77	*t* (26.52) = 18.15, *p* < .001

*Note*: *β* (*SE*) represents the mean (and standard error) of the slope for each trait. Confidence intervals (CI) are used to compare differences in the slopes, where non‐overlapping 84% CIs indicate a significant difference at *p* < .05. *T*‐tests are used to determine if the slope is significantly different from 0.

Comparing the confidence intervals of the traits (presented in Table 3), we found the size‐related modulation was significantly stronger for trustworthiness compared with the other traits. This means that people's ratings of trustworthiness were more amplified (i.e., ratings were more extreme) at implied near distance compared with the other three traits.

Table 4 details the descriptive statistics associated with ratings of large versus small images on the four targeted traits.

**TABLE 4:** Mean values and 95% confidence intervals of the four traits with Large and Small image sizes.

	Large	Small
Attractiveness	3.44 [3.33, 3.56]	3.46 [3.35, 3.58]
Competence	3.93 [3.83, 4.03]	4.02 [3.93, 4.11]
Dominance	3.75 [3.64, 3.86]	3.59 [3.48, 3.69]
Trustworthiness	3.65 [3.56, 3.75]	3.69 [3.59, 3.79]

### Discussion

In Experiment 2, we found that like changing physical viewing distance, changing stimulus size also affects impression ratings. Specifically, trait attributions of other people are amplified when tested with larger‐size images (images of people rated high/low on a trait with small images are rated even higher/lower when rated on large images). Trait ratings for trustworthiness are even more amplified than the other traits with larger sizes. These results show that size and distance have a similar modulation of trait impressions, although they affect some traits slightly differently. This could mean that some of the effects of distance are at least partially driven by perceptual information such as stimulus size. As discussed in the Introduction, much of the previous research has been using small face stimuli, which led us to compare them with larger, life‐sized images, also serving as distance proxies. The results presented here show that small image‐driven research by others could be underestimating trait eccentricity.

## EXPERIMENT 3

Experiment 3 tested whether spatial frequency information, a perceptual cue that varies with distance, could potentially contribute to the observed effects of interpersonal distance on trait attribution in Experiment 1. As distance from an image increases, high spatial frequency visual information is degraded (Lampinen et al., 2014; Loftus & Harley, 2005; McKone, 2009) and thus will be unavailable for supporting impression formation.

Previous work has shown that the amygdala responds both to highly trustworthy and untrustworthy faces both when the images contain high and low spatial frequency information Said et al. (2009). This indicates that both types of information are conveyed to the amygdala when processing faces. Silvestri et al. (2022) has shown that under some conditions, participants were able to make reliable trustworthiness judgements even when they contained only low visual spatial frequency information (see also Øvervoll et al., 2020). It is not clear from this extant work how changes in spatial frequency (as a proxy for changes in distance) would be predicted to affect perception/ratings across the four targeted traits. Together, these studies imply that there may in fact be some retention of stability of impression judgements across the spatial frequency spectrum, although our design may provide greater sensitivity to subtle differences.

To investigate the potential contribution of differences in available visual information to the results observed in Experiment 1, we contrasted trait ratings of low spatial frequency (LSF; removing the high spatial frequency information) filtered stimuli with those of standard stimuli which include full frequency spectrum or broad spatial frequency (BSF) information. We kept both stimulus size and viewing distance constant (large, 1 m) but varied spatial frequency information by blurring the images (Gaussian blur, radius 5px) in the LSF condition, which in effect emulates some aspects of the visual information available at far distances. If available spatial frequency information plays a role in the effect of distance on impressions, then we would expect to observe a similar pattern of responses to that in Experiment 1. However, if the mechanism underlying the effect of interpersonal distance is more social in nature, it may be not affected spatial frequency information, then the spatial frequency manipulation may have little effect on trait attributions.

### Method

#### Participants

Thirty‐three participants (*M* = 19.6 years, *SD* = 2.6, range from 18 to 33 years; 23 female, 8 male, 2 non‐binary) completed this experiment.

#### Apparatus and stimuli

**FIGURE 5:**
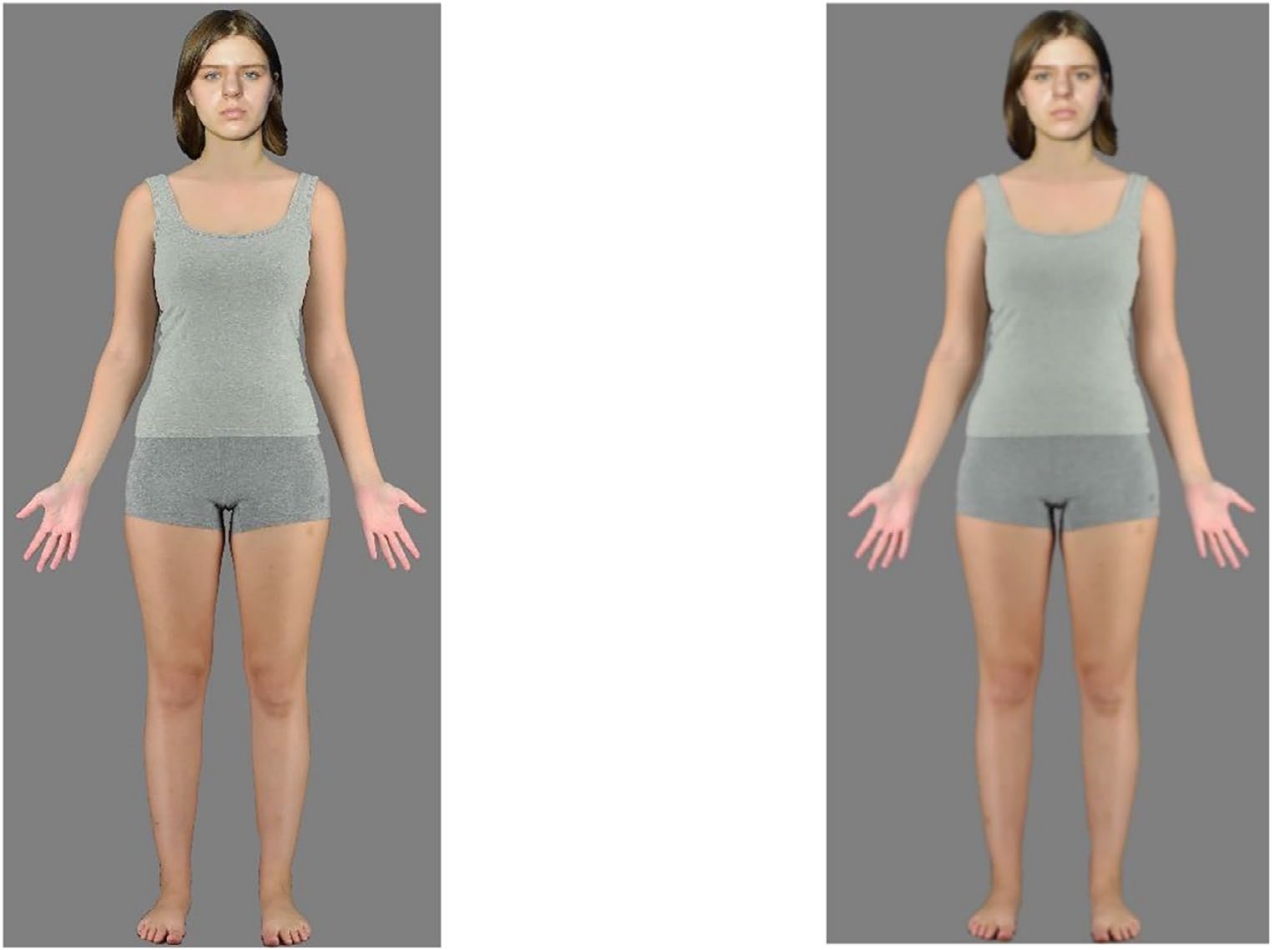
Representative examples of Broad and Low Spatial Frequency stimuli. Representative examples used in Experiment 3.

The stimulus set was the same as used in Experiments 1 and 2. The appearance of the BSF images matched those used in Experiment 1, whereas the LSF images were created with Adobe Photoshop: applying the Gaussian blur function with radius of 5px. This blur value was selected following an informal appraisal of the literature but was mainly guided by the subjective impression of the authors as delivering the required percept (for examples of stimuli see Figure 5).

#### Design and procedure

Participants stood a fixed 1 m from the projector screen and rated the test stimuli when presented as BSF images (not blurred, as in Experiment 1) and LSF images (blurred to simulate presentation at a distance) in separate blocks. All other aspects of the procedure matched Experiment 1.

### Results

We applied the same analyses reported for Experiment 1 using image spatial frequency (BSF, LSF) as proxy for distance (near, far). Both the overall model and the separate models for each of the traits indicate that as baseline stimulus ratings increase (i.e., increased ratings), the spatial frequency‐related modulation is increased for all traits i.e., people's baseline ratings of all traits were amplified (i.e., ratings were more extreme) with BSF images. This means that people's trait impression ratings made from low spatial frequency images are amplified when presented with BSF (i.e., people's ratings of BSF images were more extreme than those of low spatial frequency images). Modelling of different traits separately revealed that attractiveness ratings are selectively different to all the other traits. The estimate of attractiveness is less positive than the other traits, which means that in comparison people's impressions of attractiveness are more stable with different spatial frequencies.

The linear mixed effects modelling analysis revealed significant fixed effects of trait, F(3, 79.83) = 11.25, *p* < .001, baseline stimulus ratings, F(1, 51.49) = 535.12, *p* < .001 and their interaction, F(3, 69.96) = 16.26, *p* < .001. The model shows that spatial frequency‐related modulation of impressions is significantly predicted by trait (i.e., if someone is considering attractiveness, trustworthiness, competence or dominance), baseline stimulus ratings (i.e., the extent to which an individual stimulus has high or low ratings on the given trait irrespective of distance) and their interaction.

**FIGURE 6:**
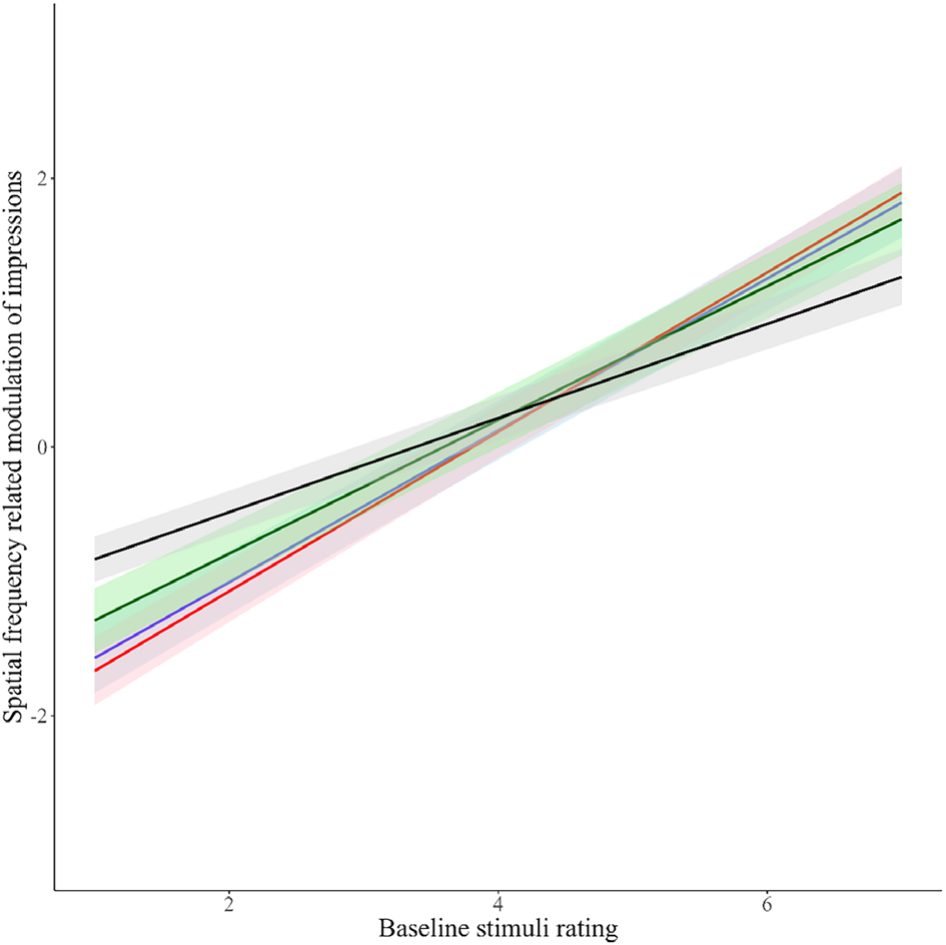
Relationship of spatial frequency‐related modulation of impressions and baseline ratings for each of the four traits. Black line = attractiveness; Red line = competence; Blue line = dominance; Green line = trustworthiness. Spatial frequency‐related modulation of impressions was calculated as BSF (broad spatial frequency) minus LSF (low spatial frequency), which means that the stimuli identities that were above the x = 0 were rated higher with BSF images and those below were rated as higher with LSF images.

Figure 6 shows the ratings of each participant of each stimulus for the four traits. It depicts the relationship between spatial frequency‐related modulation of impressions and baseline stimuli ratings. These relationships are presented numerically in Table 5. The positive slopes (*β*) indicate a positive relationship between spatial frequency‐related modulation of impressions and baseline stimuli ratings. One sample *t*‐tests show that the slope is significantly different from zero. Together, these show a substantial increase in the spatial frequency‐related modulation of impressions as baseline ratings increased. This means that adding high spatial frequencies to images amplifies first impressions (e.g., trustworthy looking people look even more trustworthy with BSF images, untrustworthy looking people look even less trustworthy with BSF images). This effect was again qualified by the interaction between trait and spatial frequency‐related modulation of impressions, showing that attractiveness was less affected by spatial frequency than all the other traits.

**TABLE 5:** Spatial frequency‐related modulation of each of the four traits.

Trait	*β*	*SE*	*84% CI*		*One sample t‐test*
			*LL*	*UL*	
Attractiveness	.35	.02	.31	.38	*t* (25.59) = 15.17, *p* < .001
Competence	.59	.02	.55	.62	*t* (24.02) = 20.76, *p* < .001
Dominance	.57	.03	.52	.60	*t* (24.38) = 17.42, *p* < .001
Trustworthiness	.50	.04	.43	.54	*t* (27.84) = 14.31, *p* < .001

*Note*: *β* (SE) represents the mean (and standard error) of the slope for each trait. Confidence intervals (CI) are used to compare differences in the slopes, where non‐overlapping 84% CIs indicate a significant difference at *p* < .05. *T*‐tests are used to determine if the slope is significantly different from 0.

Comparing the confidence intervals of the traits (presented in Table 5), we found the spatial frequency‐related modulation is different for attractiveness than for the other traits–people's ratings of attractiveness were more similar with LSF and BSF images compared with the other three traits. The positive relationship observed broadly is significantly less steep for attractiveness compared with the three other traits. Interestingly, competence was found to be more modulated by spatial frequency compared with trustworthiness.

Table 6 details the descriptive statistics associated with the ratings of LSF versus BSF images for the four targeted traits.

**TABLE 6:** Mean values and 95% confidence intervals of the four traits with broad (BSF) and low spatial frequency (LSF) images.

	BSF	LSF
Attractiveness	3.38 [3.26, 3.50]	3.36 [3.24, 3.47]
Competence	3.86 [3.75, 3.96]	4.00 [3.90, 4.10]
Dominance	3.71 [3.59, 3.82]	3.67 [3.56, 3.79]
Trustworthiness	3.67 [3.56, 3.78]	3.76 [3.65, 3.86]

### Discussion

When spatial frequency information is manipulated as a proxy for distance, we found that people's impressions of others are amplified (i.e., images of people rated high/low on a trait far away are rated even higher/lower when rated near) from low to BSF images. This effect was similar to that observed with the manipulation of physical interpersonal distance. Furthermore, as with distance manipulation, we found that ratings of attractiveness were more similar (i.e., relatively less amplified) between low and BSF images compared to the ratings of competence, dominance and trustworthiness. Therefore, distance and spatial frequency seem to share a more similar pattern than size when it comes to the modulation of attractiveness. These results indicate that changes in spatial frequency information modulate trait impressions in a very similar way to changes in distance, i.e., ratings made Near and with BSF images are more amplified compared with trait ratings made Far and with LSF. Furthermore, the correspondence between these two experiments cross‐validates our blur‐degree choice.

## EXPERIMENT 4

In this final experiment, we aimed to cross‐validate our experimental approach. One key measure in interpersonal distance research is ‘comfort distance’. This describes the minimal proximity that a person feels comfortable for another person. Our experiments so far have asked participants to make judgements about life‐sized images of people. While we used this novel approach to examine person perception, we still are only assuming that this approach holds some ecological validity. One way of assessing the degree to which we were successful in this aim is to ask participants to establish their comfort distance from an image of a person and also a real‐life person. Similar patterns of responses could serve as a source of validation of our general approach across Experiments 1–3.

We utilized a classic ‘comfort distance estimation’ task in which participants are asked to determine the distance at which they would feel comfortable standing from a target (Candini et al., 2021; Hecht et al., 2019; Iachini et al., 2014; Sorokowska et al., 2017). To establish whether participants are sensitive to the social relevance of an image projected onto a screen–as they are in ‘real life’–we measured the association between comfort distance estimates for these life‐sized stimuli and those of a real person (i.e., the experimenter). Furthermore, to establish the selectivity of any correlations, we also measured the association between the experimenter and another physically present, but non‐social control category: a dressmaking mannequin. Furthermore, it has been established that the distance people keep from others correlates with their levels of social anxiety (Givon‐Benjio & Okon‐Singer, 2020; Perry et al., 2013). We therefore included a measure of social anxiety to additionally understand how well the projected images yield similar responses across individuals with different levels of social anxiety as in real life interactions.

### Method

#### Participants

Two hundred and nine adults (*M* = 20.9 years, *SD* = 4.8 years, range from 18 to 47 years; 166 female, 40 male, 3 non‐binary) completed this experiment. These participants completed the task after taking part in Experiment 1 (*N* = 43), Experiment 2 (*N* = 24), Experiment 3 (*N* = 16) and 3 unpublished studies (*N* = 126), which all used a similar design.

#### Apparatus and stimuli

**FIGURE 7:**
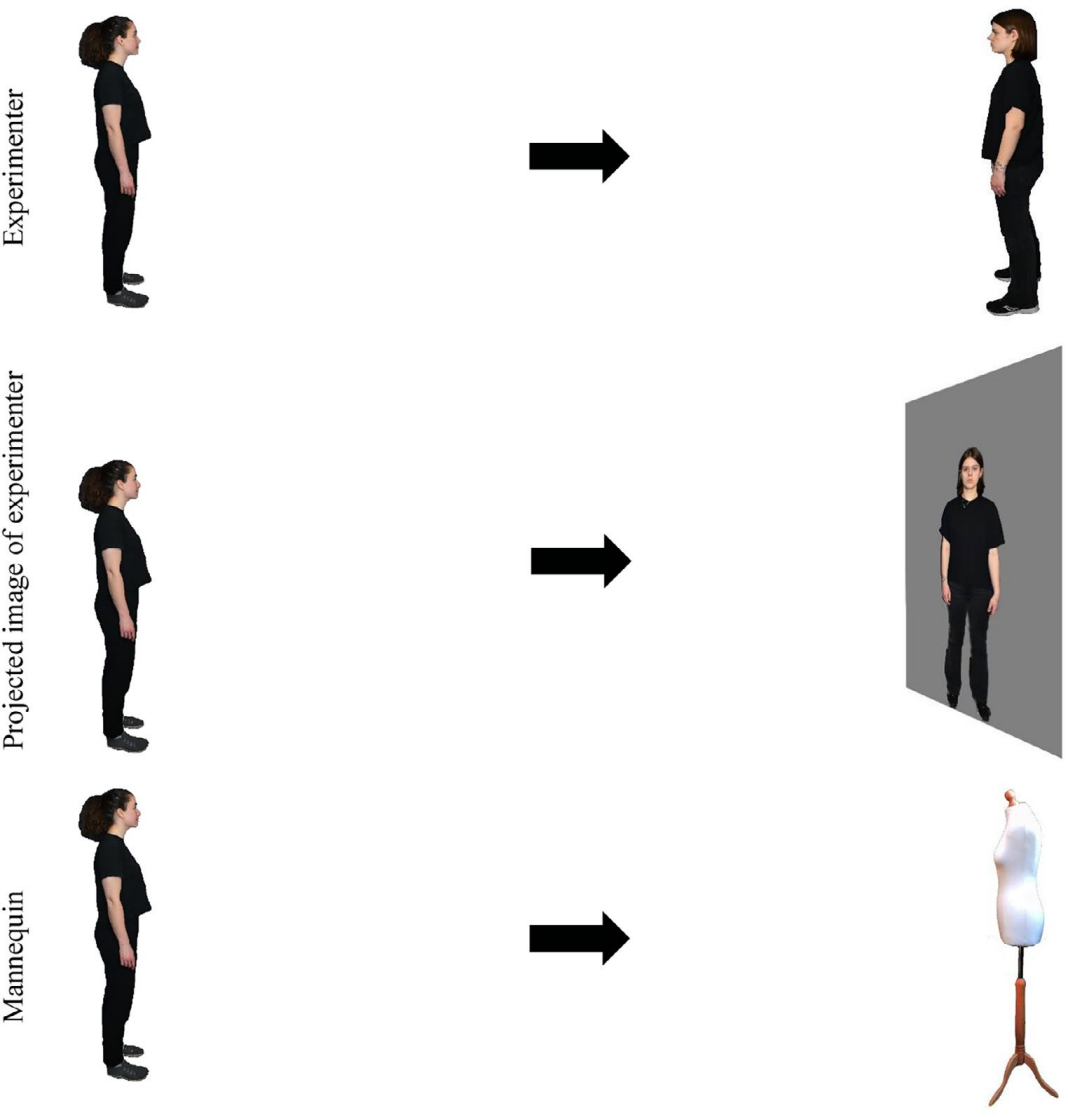
Schematic representations of comfort distance conditions of Experiment 4. Three comfort distance tasks of the participant (shown on the left of the figure) was to approach (direction of approach indicated by the arrows) the experimenter, projected image of the experimenter and the mannequin.

The same apparatus was used as in Experiments 1–3. In addition, a dressmaking mannequin (comprised a female torso with no head, arms or legs) was used as a non‐social reference point to enable the comparison of comfort distances to a person with a non‐person object that shared some stimulus features of a person (see Figure 7). The projected image was always a life‐sized image of the author KV. The image of the Experimenter and the mannequin were both 1.65 m tall. Six different experimenters measured Comfort Distances across experiments, all aged 20–27 years and identifying as European females (post‐hoc analyses confirmed no differences in distance preferences across identities, see Data S1 Section 3 for details). All experiments were conducted in a large room (11.7 m × 7.5 m × 3.8 m) with low lighting. Distances were measured with a laser measuring tool (RockSeed meter, measuring range 50 m, accuracy ±0.16 m).

#### Design and procedure

Participants took part in three comfort distance approach tasks (approaching the experimenter, an image of the experimenter and a mannequin) and completed the Brief Fear of Negative Evaluation Scale (FNEB, Leary, 1983), a well validated measure of social anxiety (Collins et al., 2005). Participants were introduced to comfort distance as a construct (i.e., the distance at which people feel comfortable standing from others) and told they would be asked to identify their comfort distance from the experimenter, a mannequin and an image of the experimenter. Using a similar procedure as in Hecht et al. (2019), we asked participants to approach each stimulus from 2.5 m and were asked to stop when they felt comfortable to be standing in relation to the person/mannequin/image, just before they would start feeling uncomfortable.

## RESULTS AND DISCUSSION

We found the comfort distances kept to experimenter, screen and mannequin to be highly correlated (all rs >.41, all ps <.001, see Table 7). In addition, we observed significant positive correlations between social anxiety and mean comfort distances kept from the real person (*r* = .19, *p* = .006) and from the projected life‐sized image of the person (*r* = .20, *p* = .004). In replicating previous research (Givon‐Benjio & Okon‐Singer, 2020; Perry et al., 2013), these results suggest that participants respond to these life‐sized projections in a similar manner to real person targets. That is, those more fearful of negative evaluation keep larger distances from both real and projected people. This finding also lays a sound foundation that life‐sized projected images can act as a valid proxy for investigating proxemic behaviour. Critically, social anxiety levels were not significantly associated with comfort distances from the mannequin (*r* = .09, *p* = .189), suggesting that such associations are relatively specific to interpersonal distances and not a generic effect associated with distance preferences. These results support that the findings from Experiment 1 found with projected images could be at least somewhat generalizable to real people. It should be noted that we collected the data for this experiment from participants who had some experience with such projected images, i.e., they had all completed projector‐based experiments, such as those described in Experiments 1–3.

**TABLE 7:** Comfort distances (in metres) and correlations between comfort distances and the scores on the Fear of Negative Evaluation Scale.

	*M* (95% CI)	1	2	3
1. FNEB	41.11 [39.64, 42.59]			
2. Comfort distance: Person	0.73 [0.69, 0.77]	.19**		
3. Comfort distance: Image	0.83 [0.78, 0.89]	.20**	.47***	
4. Comfort distance: Mannequin	0.59 [0.54, 0.63]	.09	.41***	.66***

*Note*: **indicates *p* < .01; ***p < .001. M, CI and FNEB are used to represent mean, confidence intervals and Brief Fear of Negative Evaluation Scale respectively; comfort distances reported in metres. FNEB scored 0–60.

## GENERAL DISCUSSION

The current study investigated how social perception of other people is affected by interpersonal distance–a crucial perceptual and social cue in our social interactions. Across four experiments using life‐size images of people, we showed that both physical interpersonal distance (Experiment 1) and implied interpersonal distance based on image size and visual spatial frequency information (Experiments 2 and 3) modulates how people attribute high‐level social traits to other people. Furthermore, we also showed that life‐size projection of whole person stimuli functions similarly to real life people in determining comfort distance (Experiment 4). The results of our main study (Experiment 1) revealed that ratings tended to be amplified at near compared with far distance. That is, images of individuals who were rated as less dominant (for example) were considered even less dominant at near distances. This pattern of relatively amplified responses at a near distance was true for all traits, but distance had relatively stronger effects on trustworthiness, competence and dominance compared with attractiveness judgements. This distinction may highlight differences in the distance‐related stability of the critical cues that influence more ‘social’ versus ‘aesthetic’ judgements. Our final experiment (Experiment 4) has shown that comfort distances to the person correlate with the distances kept to the image and are similarly related with social anxiety, which implies that the outcomes we observed with projected images would be similar if we used real people.

The modulation of trait judgements by physical distance was also observed with perceptual visual distance proxies–size and spatial frequency information. That is, all trait ratings were consistently enhanced (highly rated people were rated even higher and low rated people were rated even lower) when stimulus conditions were analogous to being closer to the participants (i.e., presented with BSF or in large stimulus size). Moreover, while manipulation of spatial frequency showed a similar distinction between its effects on attractiveness versus other traits, size modulation showed no such difference. These results suggest that the effect of interpersonal distance on impressions may be mediated by physical differences in stimuli, not just the difference implied social relevance of a close person compared with a distal person.

Given that perceptual information and the social importance of a given target changes with distance, we hypothesised that social attributions (i.e., trait ratings) might also vary predictably when an individual appears near versus far. Furthermore, we reasoned that the magnitude of these effects might depend on the specific characteristic under consideration. Trustworthiness, competence and dominance are traits that are related to how beneficial or harmful someone can and will be for an individual (e.g., Oosterhof & Todorov, 2008; Sutherland et al., 2013). When people make these judgements, it follows that they are considering another's potential utility and harm. Proximity might be a more salient influence upon these traits because collaboration and threat are most relevant at close distances. Furthermore, at further distances we have less detail and might make our judgements more cautiously. As a result, people might prefer to rate others closer to the average at far distances.

Another mechanism that could be involved is relative shifts in reliance on information from the face and the body. Our design did not allow us to test for this, but this could have meant that distance also has variable impacts upon different trait judgements because changes in distance shift participants' relative reliance on information in the face versus body (see Hahn et al., 2016). Hu and O'Toole (2022) identified differences in the diagnosticity of information in the face, body or whole person for different traits, e.g., trustworthiness is predominantly judged from facial information, whereas dominance also includes a contribution from the body (Hu & O'Toole, 2022). Our attractiveness results may indicate that relative to the other traits examined here, this trait is associated more strongly with cues in both the face *and* body: leaving them relatively more stable across viewing distances. The results of previous studies on the reliance of attractiveness judgements on body and face that show strong positive correlations between attractiveness ratings from bodies and faces support such an account of the current data (Honekopp et al., 2007; Thornhill & Grammer, 1999). Differential modulation of distance and distance‐related visual cues of different traits in our experiments could be due to the reliance for making judgements from the body compared to the face.

Our results suggest that the size of presentation of face and body images could influence the social perceptions and attributions that are made in a similar way to the distance of an observer from a person. This finding suggests that researchers should be conscious of the size of the images used in social perception studies. A non‐systematic review of the literature (based on the existing work on person perception and an additional search on PubMed and Google Scholar), with the following key words: “first impressions OR trait attributions OR trait judgements OR attractiveness OR competence OR dominance OR trustworthiness OR personality judgements OR facial traits OR trait inferences” resulted in 146 empirical research articles that asked participants to provide impression ratings of face or body stimuli. We found that only 21 (14%) of these papers (32 experiments) reported sufficient information to calculate the implied distance from the observer to the stimulus assuming it to be a life‐sized human (i.e. visual angle or viewing distance and size of the stimuli). Assuming an average person height of 1.70 m, face height of 0.20 m and body height 1.50 m, we found that 10 experiments using whole person stimuli simulated a mean distance of 11.7 m (range: 4.9–17.2 m). Experiments that presented only face stimuli (*N* = 14) simulated a mean distance of 3.5 m (range: 0.5–19.46 m), while experiments using body stimuli (*N* = 7) simulated a mean distance of 9.4 m (range: 1.3–18.2 m). Accordingly, based on Hall's (1966) interpersonal distance zones, the face stimuli would on average fall in the far zone of social space (1.2–3.7 m) and the body and whole person stimuli within the most distal ‘public’ space (>3.7 m; Hall, 1966) and all would have fallen far beyond the intermediate ‘social’ space according to work by Sorokowska et al. (2017), who found distances kept from strangers to be on average 1.35 m. The discrepancies between the implied interpersonal distance from stimulus size typically used in the field may not be inconsequential because the quality of social interactions differs dramatically between the implied distances in these studies and the distances at which most every day in‐person social interactions occur. Our study demonstrated that increased proximity and higher resolution visual information can amplify the bivalent magnitude of the perception of certain traits. This novel finding presents an opportunity for producing richer datasets by presenting life‐sized stimuli nearby when such information is desirable. Nevertheless, stimuli that imply an unrealistically far interpersonal distance may still be the most appropriate for certain research questions. Our findings certainly do not invalidate such previous research. Indeed, a key implication of our findings is that distal implied distances typical of the literature may underestimate impression variation across conditions, so the field may include some type II, rather than type I errors in this regard. Nevertheless, many research questions may be best answered by appreciating this aspect of ecological validity. At a minimum, clear reporting of stimulus dimensions is desirable as we move forward to help inform the development of this area of study.

Our study was not without limitations. Our experimental design was limited to two specific distances (1 m and 4 m), and we acknowledge that from an evolutionary standpoint it might be very unusual to form a first impression of someone appearing suddenly at a close distance. Furthermore, since social interactions occur at various distances our findings might not distinguish between effects specific to other interpersonal distances (see Patterson & Sechrest, 1970). Unfortunately, our study design, sample size and the number of stimuli prevented us from developing a more complex statistical model that could compare the effects of distance, size and spatial frequency. As a result, our comparisons are limited. Furthermore, in the main statistical analyses we were also unable to include measures such as comfort distances and social anxiety, which could help reveal further how individual differences affect perception of others. As noted, participants who completed Experiment 4 had also participated in either Experiment 1, 2 or 3 immediately prior to the comfort distance task of Experiment 4, which could have resulted in some carry‐over or fatigue effects. Future studies could investigate confidence ratings after participants provide trait impressions. It is possible that part of our amplification effect is due to proximity enhancing the confidence in which participants form their opinions, encouraging them to move towards the extremes of the scale. Similar to the majority of work on trait impression, we did not collect data on confidence of ratings. One study on confidence of ratings in trait impression has shown that judgement extremity was indeed a significant predictor of judgement confidence (Ames et al., 2010). It is important to note however, that the effect of ratings becoming more extreme with proximity was weaker for attractiveness judgements, and it is not clear that shifts in confidence would be less for attractiveness than for other traits. Finally, our stimulus set lacked diversity across variables such as race and age, highlighting the need for future studies to validate whether our findings are generalizable to a broader and more diverse range of stimuli.

## CONCLUSIONS

Real‐life social interactions occur in space and are shaped by interpersonal spatial information, yet few studies have considered how interpersonal distance affects social perception of others. Here, we addressed this question by using a validated and ecological paradigm using life‐size images of people in trait attribution tasks. We found that at closer distances the ratings of all impressions are amplified. That is, images of people rated high/low on a trait far away are rated even higher/lower when rated near. This result was also found for proxies of distance–size and spatial frequency. However, this amplification effect is much weaker when participants judge attractiveness. Here interpersonal distance has a much weaker modulatory effect; very (un)attractive people are rated similarly near and far. It is possible that body cues could have contributed stability to attractiveness judgements, while judgements of other traits may be more reliant on facial cues. We also found that the relationship between trait judgements and interpersonal distance can be broadly replicated by holding distance constant but manipulating low‐level visual properties of the stimuli to simulate a change in distance (i.e., size and spatial frequency). From the present study, we can conclude that personal traits are modulated by changes in interpersonal distances. Finding similar results across different distance‐related visual cues indicates that the effect is at least partly perceptually driven, in addition to the socio‐evaluative factors that are engaged when interacting with others.

## AUTHOR CONTRIBUTIONS

**Kristina Veranic:** Conceptualization; investigation; funding acquisition; writing – original draft; methodology; visualization; writing – review and editing; formal analysis; project administration; data curation. **Andrew P. Bayliss:** Conceptualization; investigation; funding acquisition; methodology; visualization; writing – review and editing; project administration; supervision; formal analysis. **Mintao Zhao:** Conceptualization; writing – review and editing; supervision; formal analysis. **Ian D. Stephen:** Investigation; visualization; writing – review and editing; formal analysis; methodology. **Louise Ewing:** Conceptualization; investigation; funding acquisition; methodology; visualization; writing – review and editing; formal analysis; supervision; project administration.

## CONFLICT OF INTEREST STATEMENT

All authors declare that they have no conflicts of interest to disclose.

## Supporting information


Data S1.

## Data Availability

The data that support the findings of this study are openly available in OSF at http://doi.org/10.17605/OSF.IO/KJ6YC
