# Supplementary material for: Close encounters: Interpersonal proximity amplifies social appraisals
Source: Br J Psychol. 2025 Feb 21;116(3):594–616. doi: 10.1111/bjop.12781 (PMC12256726; doi:10.1111/bjop.12781)
Supplement: Supplementary file 1 — Data S1. [file BJOP-116-594-s001.docx]

**Supplementary materials**

**Section 1. Stimuli selection procedure**

| **Table S1,**  *Online ratings associated with the high and low trait exemplars in the four final stimulus sets (rating scale 1-5)* | | | | | |
| --- | --- | --- | --- | --- | --- |
| Trait | Category | M | SD | 95% CI | t-test |
| Attractiveness | low | 1.63 | .21 | [1.50, 1.76] | *t*(22) = -12.25, *p* < .001, *d =* 5.00 |
|  | high | 2.73 | .23 | [2.58, 2.88] |  |
| Competence | low | 2.61 | .21 | [2.48, 2.74] | *t*(12.03) = -8.05, *p* < .001, *d =* 3.27 |
|  | high | 3.11 | .05 | [3.08, 3.14] |  |
| Dominance | low | 2.15 | .27 | [1.98, 2.32] | *t*(22) = -16.14, *p* < .001, *d =* 6.62 |
|  | high | 3.62 | .16 | [3.52, 3.72] |  |
| Trustworthiness | low | 1.54 | .34 | [1.32, 1.76] | *t*(15.46) = -14.94*, p* < .001, *d =* 6.05 |
|  | high | 3.13 | .15 | [3.03, 3.23] |  |
| *Note*. In the Experiments 1-3 rating scale used was 1-7. | | | | | |

From the available dataset of images (Stephen et al., 2016) we selected high and low-rated exemplars for trustworthiness, dominance, competence and attractiveness. These were based on ratings that we collected from a sample of participants who did not complete the experiments reported in the manuscript. Specifically, seventy-two participants aged 18 to 26 years (M = 20.7 years, SD = 3.7 years; 64 female, 8 male, 0 non-binary) rated 159 images for dominance, trustworthiness, competence, or attractiveness (between subjects, each trait was rated by 18 participants). Assuming a standard viewing distance (60cm) images appeared with a visual angle of approximately 17° visual angle. Participants rated the stimuli from 1 to 5 on a Likert-like scale. The mean ratings for each stimulus were then used to manually select four non-overlapping stimulus sets for use in the Experiments reported in the manuscript. Our goal was to ensure large numerical differences between those stimuli rated low and high on each given trait, while also balancing the need to find extreme exemplars for other traits (i.e., we did not necessarily allocate all the highest attractive images to the attractiveness condition, because some of those individuals were also rated highly on trustworthiness and we needed highly-rated exemplars in that trait category too). Table S1 contains descriptive statistics for low and high bins of stimuli selected for each of the traits. Included are also t-tests comparing low and high bins for each of the traits.

**Section 2. How did participant arousal change with distance from projected test stimuli?**

To validate design elements of our main experiment, we sought evidence of variability in the social salience of our projected person images when viewed at specific near versus far distances. Levels of arousal are typically higher when standing close to a (real) person compared with further away (e.g., Candini et al., 2021; Evans & Wener, 2007). We sought to replicate this pattern with participants’ ratings of emotional arousal in response to our projected stimuli and chosen ‘interpersonal distances’, to confirm that the social contingencies in our lab-based simulation corresponded with those observed in other studies including those with more ecological approaches involving real interactions with people.

***Methods***

**Participants.** Twenty-four participants (M = 19.3 years, SD = 1.2, range from 18 to 22 years; 19 female, 5 male, 0 non-binary) completed the study. These participants had all also first completed Experiment 3b reported in the main text.

**Stimuli and Apparatus.**

The stimuli consisted of high resolution (1794 x 4494 pixels) images of 96 adults of different ages and ethnic backgrounds from an existing database (for more detail see Stephen et al., 2016). Individuals are pictured wearing standard close-fitting grey singlets and shorts and facing forward in a standard posture - with their arms by their side - and a neutral facial expression (Figure 2). Each image was positioned on a grey background, so that the individual appeared to be approximately standing on the same ground plane. They appeared at realistic life sizes: males at a standard UK average height of 175cm, and females 160cm. Images were presented using the same projector setup as in Experiment 1a. Near-distance images subtended vertically 84° of visual angle, and far-distance images 25° when participants stand from 1 and 4 meters away respectively.

**Design & Procedure.** Participants were asked to rate their emotional arousal while viewing images of people on a screen at a distance of 1 meter (near) and 4 meters (far). Images appeared for 2500ms; as soon as the image appeared, the participants could take as long as they needed to make their rating using a Likert scale (ranging from 1, meaning bored/calm – 7, meaning excited/alert). They were asked to use the whole range of the scale. Their rating was followed by a 500ms interstimulus interval (ISI). Responses were made using a mouse, which rested on a platform that was moved with them to the different rating locations. Order of standing distances was counterbalanced across participants.

**Results and Discussion**

We ran item- and subject-level analyses to understand both how participants rated the stimuli at different distances and how the stimuli identities were rated at different distances. Both analyses indicated that participants’ emotional arousal ratings were significantly higher when the participants were standing near (item level: 3.13 [2.99, 3.26]; subject level: 3.13 [2.86, 3.39]) compared with when they were standing far from the projector (item level: 2.88 [2.76, 3.00]; subject level: 2.88 [2.59, 3.18]), items analysis: t(95) = 6.03, p < .001, subject analysis: t(23) = 2.67, p < .014. Greater levels of arousal at near distances are consistent with distance-related modulation of arousal responses to real people (Candini et al., 2021; Evans & Wener, 2007; Ferri et al., 2013), providing further evidence that space information plays a critical role in social perception. People closer to us are perceived as more behaviourally relevant, because we are more likely to interact with them and their actions bear more consequences for us. This finding also confirms that, just like seeing people in real life, viewing life-sized projected images of people can elicit higher arousal when they appear at a near than at a farther away distance.

**Section 3. Effect of Experimenter identity on Comfort distance judgements in Experiment 4**

In Experiment 4, six Experimenters were measuring participants’ comfort distances. These were all white European females aged 20-27 years (author KV and AJ, FC, KS, TP, and TR mentioned in the acknowledgements). We tested whether there were differences between the experimenter identities in the comfort distances kept to each person. We found no significant difference associated with experimenter identity, F(5, 203) = 1.24, p = .292, f^2^ = .50.
